# Brainstem volume mediates seasonal variation in depressive symptoms: A cross sectional study in the UK Biobank cohort

**DOI:** 10.1038/s41598-020-60620-3

**Published:** 2020-02-27

**Authors:** Naif A. Majrashi, Trevor S. Ahearn, Gordon D. Waiter

**Affiliations:** 1 Aberdeen Biomedical Imaging Centre, University of Aberdeen, Aberdeen, UK; 2 Medical Physics, NHS Grampian, Aberdeen, UK; 3 Diagnostic Radiology Department, College of Applied Medical Sciences, Jazan University, Jazan, Saudi Arabia

**Keywords:** Brain, Magnetic resonance imaging

## Abstract

Seasonal differences in mood and depressive symptoms affect a large percentage of the general population, with seasonal affective disorder (SAD) representing the most common presentation. SAD affects up to 3% of the world’s population, and it tends to be more predominant in females than males. The brainstem has been shown to be affected by photoperiodic changes, and that longer photoperiods are associated with higher neuronal density and decreased depressive-like behaviours. We predict that longer photoperiod days are associated with larger brainstem volumes and lower depressive scores, and that brainstem volume mediates the seasonality of depressive symptoms. Participants (N = 9289, 51.8% females and 48.1% males) ranging in age from 44 to 79 years were scanned by MRI at a single location. Photoperiod was found to be negatively correlated with low mood and anhedonia in females while photoperiod was found to be positively correlated with brainstem volumes. In females, whole brainstem, pons and medulla volumes individually mediated the relationship between photoperiod and both anhedonia and low mood, while midbrain volume mediated the relationship between photoperiod and anhedonia. No mediation effects were seen in males. Our study extends the understanding of the neurobiological factors that contribute to the pathophysiology of seasonal mood variations.

## Introduction

Seasonal fluctuations in mood and depressive symptoms affect a large number of the general population, and these depressive symptoms such as depressed mood and fatigue have been found to be greater in winter compared to summer seasons in higher latitude countries^1–4^. Populations with seasonal affective disorder (SAD), a type of recurring major depression with a seasonal pattern, represent the most common form of seasonal fluctuations in mood^5,6^. SAD is often characterized by depression and fatigue occurring in winter with full remission taking place in summer. It has been reported that SAD affects up to 3% of the world’s population, and it tends to be more predominant in females than males with a reported female-to-male ratio of 4:1^7–9^. Females have been found to suffer from mood changes and depressive symptoms related to dark and cloudy weather at a greater rate compared to males^4,9^. Although seasonal variations in mood have been studied widely among sexes, little is known about the neurobiological factors linking light exposure and mood.

It has been suggested that changes in photoperiod (duration of sunlight) may be associated with seasonal mood variations^10,11^ by shifting the circadian phase with its associated disruptions in sleep and other health outcomes. However, photoperiodic changes have also been suggested to affect specific brain regions that might be implicated in mood disorders. For example, the hippocampus and hypothalamus have been shown to be affected by seasonal changes in photoperiod. In particular, a smaller volume of the hippocampus was associated with shorter photoperiods in the winter months compared to summer^12–14^, and higher gene expression and hormonal activity of the hypothalamus were associated with longer photoperiod days in summer compared to winter^15^. In addition, the brainstem has been shown to be associated with seasonal changes. In particular, in Rana temporaria L., the size of the nuclei of the medulla oblongata cells controlling lipofuscin in pigment was significantly associated with changes in photoperiod during the annual cycle, and that higher volume of the nuclei of the medulla oblongata was detected in July and lower volume was detected in March^16^. Moreover, photoperiodic changes have been shown to influence the midbrain dorsal raphe serotonin neurons. For example, mice exhibit increased firing rates, levels of mood neurotransmitters (serotonin and norepinephrine), and responsiveness to noradrenergic stimulation when exposed to longer photoperiod days compared to those exposed to shorter photoperiod days^17^. Furthermore, in humans, longer photoperiod days were significantly associated with a higher density of dopamine neurons, tyrosine hydroxylase TH (the rate limiting enzyme in dopamine synthesis) neurons, dopamine transporter (DAT) neurons, and DAT and TH neurons immunoreactivities in the midbrain compared to short photoperiod days^18^. It has been suggested that the different densities of dopamine and TH neurons could be due to neurogenesis, a new generation of neuron cells, in the brain, including midbrain^18–21^. Together, these studies suggest that changes in photoperiod directly affect the volume of the medulla oblongata, or affect the density of serotonergic and dopaminergic midbrain neurons, which in turn may lead to morphology changes of the brainstem. Therefore, we hypothesize that longer photoperiod days are associated with larger brainstem substructure volumes and shorter photoperiod days are associated with smaller brainstem substructure volumes.

Changes in the brainstem substructure volumes have been found to be linked to the pathophysiology of several mood disorders^22–24^. Photoperiodic changes in the brainstem substructures may contribute to seasonally occurring phenotypes such as seasonal affective disorder (SAD) where symptoms such as depression and fatigue are present in the winter season with full remission taking place in the summer season^2,5,25–28^. Phenotypical and morphological brainstem, particularly midbrain, changes in adult mice exposed both prenatally and postnatally to longer photoperiods are associated with decreased depressive-like and anxiety-like behaviours (by decreasing immobility during the forced swim test and time spent in the close arms of the elevated zero maze) compared to those exposed to short photoperiods^17,29^. Overall, this evidence suggests that seasonal changes in brainstem substructures are linked to depressive-like behaviours.

In light of this, a cross-sectional study within a large population cohort, was conducted to analyse the links between seasonal variation in photoperiod with seasonal variation in mood and seasonal variation in brainstem volume. The aim was to explore whether seasonal variation in depressive symptoms including low mood, anhedonia, tenseness and tiredness was mediated by brainstem or substructure volumes.

## Results

### Participant characteristics

Nine thousand and two hundred and eighty-nine participants (51.8% females, 48.1% males ages ranging from 44 and 79 years (mean = 62.4, SD = 7.4)) taken from the UK biobank cohort were included in this study. High resolution three-dimensional T1 weighted images were collected from all participants. The MRI scans were acquired between May 2014 and December 2016 with the date of scan recorded for each participant. All 9,289 participants completed a touchscreen questionnaire on their mood in the two weeks prior to the MRI assessment. Participants lived in approximately equal proportions north and south of the scanning centre with a mean distance of 31.1 km North or South. Photoperiod for each participant’s date of scan was measured based on the location of residence of each participant. The average range of observed photoperiod is from 7.25 hours in winter to 17.22 hours in summer. The range for each mood measure was from 0 to 3 (where 0 = not at all and 3 = nearly every day). Demographic characteristics are presented in Table 1.

**Table 1:** Characteristics of the UK Biobank participants.

Variable	Mean (SD)
Number of all participants	9289
Age (years)	62.4 (7.4)
Low mood	0.21 (0.50)
Anhedonia	0.19 (0.45)
Tenseness	0.24 (0.52)
Tiredness	0.57 (0.74)
Total depressive score	1.21 (1.76)
Townsend deprivation score	−2.01 (2.56)
Number of females	4817
Age (years)	61.7 (7.2)
Low mood	0.24 (0.54)
Anhedonia	0.20 (0.51)
Tenseness	0.26 (0.54)
Tiredness	0.65 (0.78)
Total depressive score	1.35 (1.87)
Townsend deprivation score	−1.96 (2.57)
Number of males	4472
Age (years)	63.1 (7.5)
Low mood	0.17 (0.45)
Anhedonia	0.18 (0.47)
Tenseness	0.22 (0.50)
Tiredness	0.49 (0.68)
Total depressive score	1.07 (1.63)
Townsend deprivation score	−2.05 (2.55)
**Living area, N (%)**	
Rural	671 (7.22)
Urban	8553 (92.07)
**Ethnic background, N (%)**	
White	8728 (93.96)
Black	29 (0.31)
Mixed	271 (2.91)
Asian	191 (2.05)
Chinese	30 (0.32)
Other	40 (0.43)
Photoperiod in hours	13.003 (3.05)
**Brain volumes**	
Whole brainstem (mm^3^)	25341.6 (3008)
Midbrain (mm^3^)	6026.7 (645)
Pons (mm^3^)	14750.3 (1916)
Medulla (mm^3^)	4321.4 (565)
WMV (cm^3^)	498.57 (59.3)
GMV (cm^3^)	628.04 (55.03)
TBV (cm^3^)	1125.92 (109.43)

Abbreviations: SD; Standard Deviation; N: number; WMV; White Matter Volume, GMV; Grey Matter Volume, TBV; Total Brain Volume.

### Tests of seasonality of brainstem substructure volumes using cosinor models

To test for seasonality, we used a cosinor analysis to examine brainstem substructure volumes. We found significant cosinor terms for all brainstem volumes including midbrain, pons, medulla and whole brainstem in all participants, and when separated into females and males, p < 0.001 (See Table 2). The acrophase peak (greatest volume) for the midbrain, pons, medulla and whole brainstem volumes occurred in July for all participants and males while in June for females.

**Table 2:** Cosinor parameters for brainstem substructure volumes in all participants, females and males.

Outcome	Multivariate generalized regression estimates						∆AIC	Acrophase	Amplitude
	Cosine coefficient b	(SE)	P	Sine coefficient b	(SE)	P		(Month)	
**a) All participants**									
Midbrain (n = 9289)	−67.61	6.19	<0.001	26.92	6.19	<0.001	−127.26	July	72.7
Pons (n = 9289)	−255.05	12.81	<0.001	94.11	21.37	<0.001	−143.89	July	271.8
Medulla (n = 9289)	−115.98	7.16	<0.001	43.30	7.02	<0.001	−277.9	July	123.8
Brainstem (n = 9289)	−441.75	32.76	<0.001	166.20	32.10	<0.001	−193.01	July	471.9
**b) Females**									
Midbrain (n = 4817)	−57.93	7.82	<0.001	31.27	7.63	<0.001	−62.74	June	65.8
Pons (n = 4817)	−251.40	28.58	<0.001	120.82	27.92	<0.001	−85.71	June	278.9
Medulla (n = 4817)	−110.33	9.40	<0.001	50.40	9.18	<0.001	−151.80	June	121.3
Brainstem (n = 4817)	−422.66	42.60	<0.001	204.09	41.61	<0.001	−110.05	June	469.3
**c) Males**									
Midbrain (n = 4472)	−85.15	9.43	<0.001	24.14	9.26	0.009	−80.48	July	88.5
Pons (n = 4472)	−265.05	33.17	<0.001	68.30	32.56	0.036	−61.70	July	273.7
Medulla (n = 4472)	−125.91	10.80	<0.001	36.83	10.61	0.001	−136.48	July	131.2
Brainstem (n = 4472)	−479.60	49.89	<0.001	131.53	48.97	0.007	−91.40	July	497.3

Generalized linear regression coefficients (b), robust standard error (SE), probability of significance (p) for cosine and sine transformations of the month of scan for: (a) all participants, (b) females and (c) males. Models were adjusted for age and TBV. AIC values refer to the differences between this model and a model excluding sine and cosine terms but including all the covariates (age and TBV). Seasonality of outcome variables (whole brainstem and substructure volumes except SCP) is inferred from (1) the significance (p < 0.05) of the cosine and sine generalized linear regression coefficients and (2) improved model fit included cosinor terms by reduced AIC value (∆AIC).

### Association of photoperiod with depressive symptoms

Negative binomial regression was conducted to investigate the association between photoperiod and depressive symptoms including low mood, anhedonia, tenseness, tiredness and total depressive score. For all participants there was a significant negative correlation between photoperiod and low mood (p < 0.05). When corrected for age, ethnicity, living area (urban or rural) and Townsend deprivation index the correlation in low mood did not remain significant (See Table 3). No significant correlations between photoperiod and anhedonia, tenseness, tiredness and total depressive score for all participants were seen. In females, photoperiod was negatively correlated only with low mood and anhedonia (p = 0.03) when corrected for age, ethnicity, living area (urban or rural) and Townsend deprivation index. When the p-value was Bonferroni corrected (0.05/15 = 0.003), there were no significant correlations between photoperiod and low mood and anhedonia in females. In males, no significant correlations between photoperiod and any of these depressive symptoms (low mood, anhedonia, tenseness, tiredness and total depressive score) were seen either before or after correction for the above confounders.

**Table 3:** Associations between photoperiod and all depressive symptoms in all participants, females and males.

	Model		
	b (SE)	IRR	p
**All participants**			
Low mood (n = 9289)	−0.015 (0.008)	0.984	0.053
Anhedonia (n = 9289)	−0.013 (0.008)	0.987	0.122
Tenseness (n = 9289)	−0.007 (0.007)	0.993	0.339
Tiredness (n = 9289)	0.002 (0.005)	1.002	0.700
Total depressive score (n = 9289)	−0.005 (0.004)	0.995	0.247
**Females**			
Low mood (n = 4817)	−0.022 (0.010)	0.978	**0.038**
Anhedonia (n = 4817)	−0.025 (0.011)	0.975	**0.033**
Tenseness (n = 4817)	−0.014 (0.010)	0.986	0.175
Tiredness (n = 4817)	0.000 (0.007)	1.000	0.980
Total depressive score (n = 4817)	−0.010 (0.006)	0.990	0.111
**Males**			
Low mood (n = 4472)	−0.009 (0.012)	0.991	0.487
Anhedonia (n = 4472)	0.001 (0.012)	1.001	0.920
Tenseness (n = 4472)	0.000 (0.011)	1.000	0.985
Tiredness (n = 4472)	0.003 (0.008)	1.003	0.721
Total depressive score (n = 4472)	−0.001 (0.006)	0.999	0.914

Negative binomial regression coefficients (b), robust standard error (SE) and incidence rate ratios (IRR) for association between photoperiod (corrected for age, ethnicity, living area and Townsend deprivation index) and low mood, anhedonia, tenseness, tiredness and total depressive score. Significant associations (p < 0.05) are shown in bold.

### Association of brainstem substructure volumes with photoperiod

**Figure 1:**
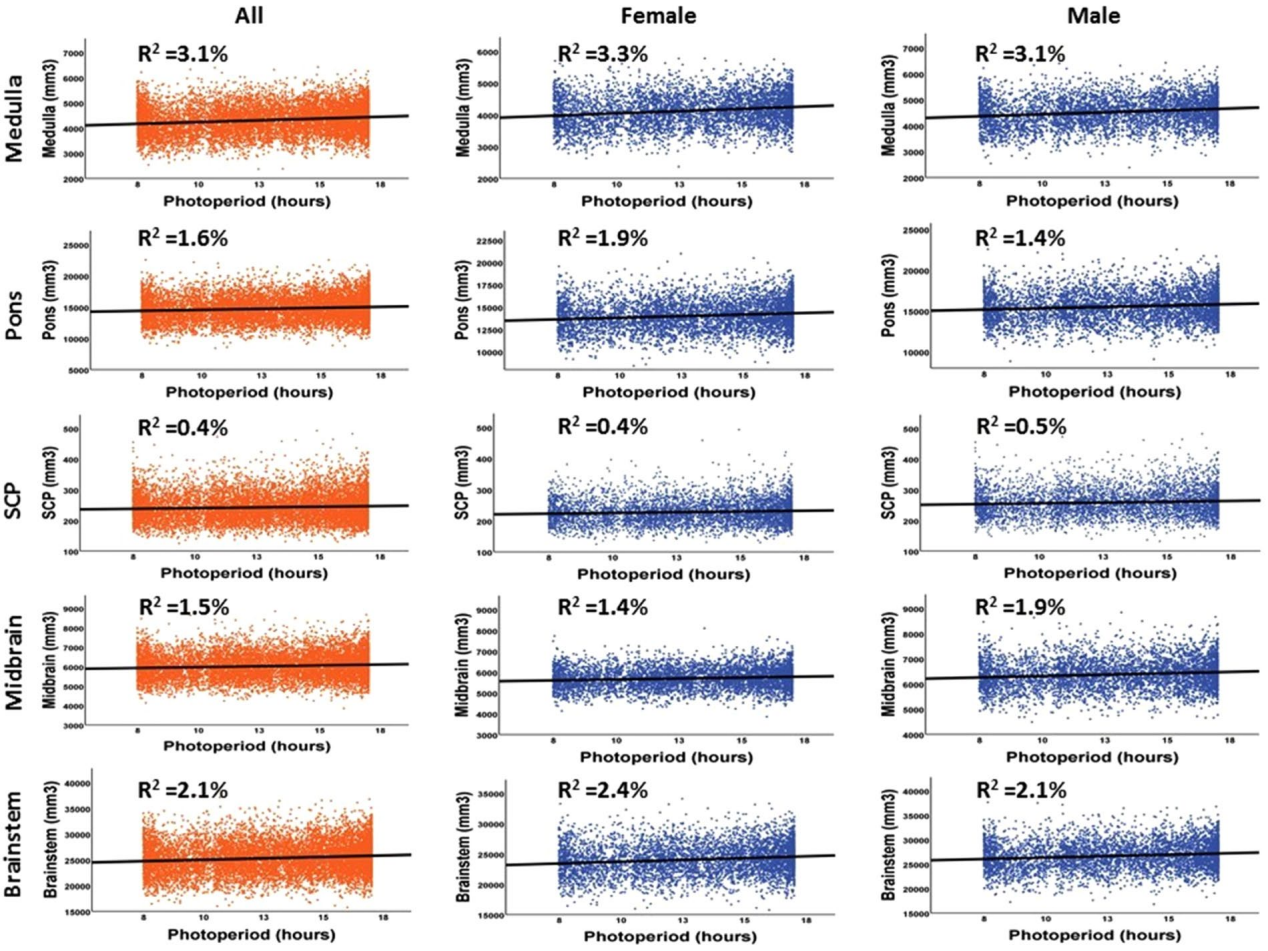
Linear correlations between brainstem substructure volumes and photoperiod.

There were significant correlations between brainstem and substructure volumes and photoperiod in all participants and both females and males separately when the p-value was corrected for multiple comparisons (p < 0.003). To further explore the observed association between brainstem volume and photoperiod, a multiple linear regression model was applied to better understand the variance in the brainstem volume when accounting for known confounds. Age and total brain volume (TBV) covariates were found to associate with brainstem and substructure volumes, and that age was negatively correlated with all brainstem volumes (p < 0.001), while TBV was positively correlated with all brainstem volumes in all participants and both females and males. Thus, these covariates were entered in separate blocks in a hierarchical regression model to account for their confounding effects. When photoperiod was corrected for these two covariates, there were significant correlations between brainstem and substructure volumes and photoperiod in all participants and both females and males separately with the p-value corrected for multiple comparisons (p < 0.003). Photoperiod was positively correlated with whole brainstem r^2^(9289) = 0.021, medulla r^2^(9289) = 0.031, pons r^2^(9289) = 0.016, SCP r^2^(9289) = 0.004 and midbrain r^2^(9289) = 0.015 volumes in all participants, and with whole brainstem r^2^(4817) = 0.024, medulla r^2^(4817) = 0.033, pons r^2^(4817) = 0.019, SCP r^2^(4817) = 0.004 and midbrain r^2^(4817) = 0.014 volumes in females and with whole brainstem r^2^(4472) = 0.021, medulla r^2^(4472) = 0.031, pons r^2^(4472) = 0.014, SCP r^2^(4472) = 0.005 and midbrain r^2^(4472) = 0.019 volumes, p < 0.001 in males (Fig. 1 and Table 4).

**Table 4:** Linear correlations between brainstem substructure volumes (corrected for age and total brain volume) and photoperiod in all participants, females and males.

	Volume M (SD)	r	B (SE)	p
**All participants (n = 9224)**				
Whole brainstem	25341.6 (3008)	0.147	105.6 (7.3)	<0.001
Midbrain	6026.7 (645)	0.123	16.5 (1.3)	<0.001
SCP	243.13 (44)	0.064	0.807 (0.13)	<0.001
Pons	14750.3 (1916)	0.127	60.7 (4.9)	<0.001
Medulla	4321.4 (565)	0.175	27.5 (1.6)	<0.001
**Females (n = 4817)**				
Whole brainstem	24086.9 (2547)	0.155	104.5 (9.5)	<0.001
Midbrain	5701.1 (507)	0.120	14.7 (1.7)	<0.001
SCP	228.27 (38)	0.064	0.742 (0.16)	<0.001
Pons	14023.9 (1667)	0.138	62.0 (6.4)	<0.001
Medulla	4133.5 (500)	0.181	27.0 (2.1)	<0.001
**Males (n = 4472)**				
Whole brainstem	26693.1 (2879)	0.146	110.6 (11.1)	<0.001
Midbrain	6377.5 (592)	0.140	20.0 (2.1)	<0.001
SCP	259.13 (45)	0.069	0.933 (0.20)	<0.001
Pons	15532.7 (1859)	0.121	60.7 (7.4)	<0.001
Medulla	4523.7 (561)	0.176	28.9 (2.4)	<0.001

Abbreviations; SCP: Superior Cerebellar Peduncle; r: Pearson correlation (standardized regression coefficient); B: regression coefficient (mm3/hour); SE: standard error; M: mean; SD: standard deviation; p: probability of significance.

### Association of brainstem substructure volumes with depressive symptoms

There were significant correlations between brainstem and substructure volumes and depressive symptoms including low mood, anhedonia and total depressive symptoms in all participants but only in females when the group was categorised by sex (corrected for age, TBV, ethnicity, living area and Townsend deprivation index). For all participants, low mood, anhedonia and total depressive score were negatively correlated with all brainstem volumes except the midbrain and SCP, p < 0.05 for all (See Table 5). When the p-value was Bonferroni corrected (0.05/75 = 0.0006), only the correlation between medulla and total depressive symptom remained significant (p < 0.0006). In females, whole brainstem and pons were associated with low mood, anhedonia and total depressive score, and the medulla volume was associated with low mood, anhedonia, tenseness and total depressive score, whereas midbrain volume was significantly associated with only anhedonia, p < 0.05 for all (See Table 5). When the p-value was Bonferroni corrected (0.05/75 = 0.0006), the correlations between anhedonia and whole brainstem and pons remained significant (p < 0.0006). No significant correlations between SCP volume and all depressive symptoms including low mood, anhedonia, tenseness, tiredness and total depressive score were seen in females. No significant correlations between all brainstem substructure volumes and low mood, anhedonia, tenseness, tiredness and total depressive score were seen in males.

**Table 5:** Associations between brainstem substructure volumes and depressive symptoms in all participants, females and males.

	Low mood		Anhedonia		Tenseness		Tiredness		Total depressive score	
	b (SE)	p	b (SE)	p	b (SE)	p	b (SE)	p	b (SE)	p
**a) All participants**										
Midbrain (n = 9289)	−1.21 × 10^−3^ (6.2 × 10^−5^)	0.054	−7.08 × 10^−5^ (6.3 × 10^−5^)	0.265	−7.51 × 10^−5^ (5.8 × 10^−5^)	0.196	−1.70 × 10^−5^ (4.2 × 10^−5^)	0.685	−5.36 × 10^−5^ (3.4 × 10^−5^)	0.118
SCP (n = 9289)	2.5 × 10^−2^ (7 × 10^−3^)	0.705	4 × 10^−3^ (7 × 10^−3^)	0.554	0.001 (6 × 10^−3^)	0.377	1.1 × 10^−3^ (5 × 10^−3^)	0.814	2.6 × 10^−3^ (4 × 10^−3^)	0.472
Pons (n = 9289)	−3.71 × 10^−5^ (1.7 × 10^−5^)	**0.033**	−5.17 × 10^−5^ (1.8 × 10^−5^)	**0.004**	−2.08 × 10^−5^ (1.6 × 10^−5^)	0.205	−2.85 × 10^−6^ (1.1 × 10^−5^)	0.809	−1.95 × 10^−5^ (9.6 × 10^−6^)	**0.043**
Medulla (n = 9289)	−1.64 × 10^−3^ (5.2 × 10^−5^)	**0.002**	−1.55 × 10^−3^ (5.3 × 10^−5^)	**0.004**	−1.35 × 10^−3^ (4.9 × 10^−5^)	0.008	−6.46 × 10^−5^ (3.5 × 10^−5^)	0.071	−1.06 × 10^−3^ (2.9 × 10^−5^)	**<0.001**
Brainstem (n = 9289)	−2.84 × 10^−5^ (1.1 × 10^−5^)	**0.014**	−3.26 × 10^−5^ (1.2 × 10^−5^)	**0.006**	−1.80 × 10^−5^ (1.1 × 10^−5^)	0.098	−4.93 × 10^−6^ (7.8 × 10^−6^)	0.530	−1.54 × 10^−5^ (6.4 × 10^−6^)	**0.016**
**b) Females**										
Midbrain (n = 4817)	−1.53 × 10^−3^ (8.7 × 10^−5^)	0.081	−2.19 × 10^−3^ (79.6 × 10^−5^)	**0.023**	−8.05 × 10^−5^ (8.6 × 10^−5^)	0.350	−1.77 × 10^−5^ (6.1 × 10^−5^)	0.772	−7.93 × 10^−5^ (5.1 × 10^−5^)	0.119
SCP (n = 4817)	4.3 × 10^−3^ (9 × 10^−3^)	0.643	−4.7 × 10^−4^ (1.0 × 10^−3^)	0.647	0.001 (9 × 10^−3^)	0.206	7.12 × 10^−5^ (7 × 10^−3^)	0.914	3.0 × 10^−3^ (5 × 10^−3^)	0.582
Pons (n = 4817)	−6.83 × 10^−5^ (2.4 × 10^−5^)	**0.004**	−9.28 × 10^−5^ (2.6 × 10^−5^)	**<0.001**	−3.99 × 10^−5^ (2.3 × 10^−5^)	0.090	−6.67 × 10^−6^ (1.6 × 10^−5^)	0.690	−3.52 × 10^−5^ (1.3 × 10^−5^)	**0.012**
Medulla (n = 4817)	−2.36 × 10^−3^ (7.1 × 10^−5^)	**0.001**	−2.60 × 10^−3^ (7.8 × 10^−5^)	**0.001**	−1.66 × 10^−3^ (7.1 × 10^−5^)	**0.019**	−7.65 × 10^−5^ (5.0 × 10^−5^)	0.128	−1.43 × 10^−3^ (4.1 × 10^−5^)	**0.001**
Brainstem (n = 4817)	−4.71 × 10^−5^ (1.6 × 10^−5^)	**0.003**	−6.17 × 10^−5^ (1.7 × 10^−5^)	**<0.001**	−2.83 × 10^−5^ (1.5 × 10^−5^)	0.071	−7.31 × 10^−6^ (1.1 × 10^−5^)	0.513	−2.53 × 10^−5^ (9.3 × 10^−6^)	**0.007**
**c) Males**										
Midbrain (n = 4472)	4.94 × 10^−5^ (9.1 × 10^−5^)	0.588	5.45 × 10^−5^ (8.7 × 10^−5^)	0.534	2.99 × 10^−5^ (8.1 × 10^−5^)	0.713	7.02 × 10^−5^ (6.0 × 10^−5^)	0.243	4.01 × 10^−5^ (4.8 × 10^−5^)	0.407
SCP (n = 4472)	0.001 (0.001)	0.378	0.001 (0.009)	0.225	1.60 × 10^−3^ (9 × 10^−3^)	0.225	0.001 (6 × 10^−3^)	0.389	0.001 (5 × 10^−3^)	0.293
Pons (n = 4472)	9.37 × 10^−6^ (2.5 × 10^−5^)	0.715	−1.67 × 10^−5^ (2.4 × 10^−5^)	0.498	3.79 × 10^−7^ (2.2 × 10^−5^)	0.986	−8.41 × 10^−6^ (1.6 × 10^−5^)	0.618	1.31 × 10^−6^ (1.3 × 10^−5^)	0.923
Medulla (n = 4472)	−2.01 × 10^−5^ (7.8 × 10^−5^)	0.798	−5.58 × 10^−5^ (7.5 × 10^−5^)	0.458	−8.33 × 10^−5^ (7.0 × 10^−5^)	0.234	−2.08 × 10^−5^ (5.1 × 10^−5^)	0.686	−3.91 × 10^−5^ (4.1 × 10^−5^)	0.343
Brainstem (n = 4472)	5.08 × 10^−6^ (1.7 × 10^−5^)	0.765	−7.74 × 10^−6^ (1.6 × 10^−5^)	0.636	−4.77 × 10^−6^ (1.5 × 10^−5^)	0.098	5.33 × 10^−6^ (1.1 × 10^−5^)	0.634	2.68 × 10^−7^ (8.9 × 10^−6^)	0.976

Negative binomial regression coefficients (b), robust standard error (SE) for association between brainstem substructure volumes (corrected for age, ethnicity, TBV, living area and Townsend deprivation index) and low mood, anhedonia, tenseness, tiredness and total depressive score in (a) all participants, (b) females and (c) males. Significant associations (p < 0.05) are shown in bold.

### Mediation analysis

**Figure 2:**
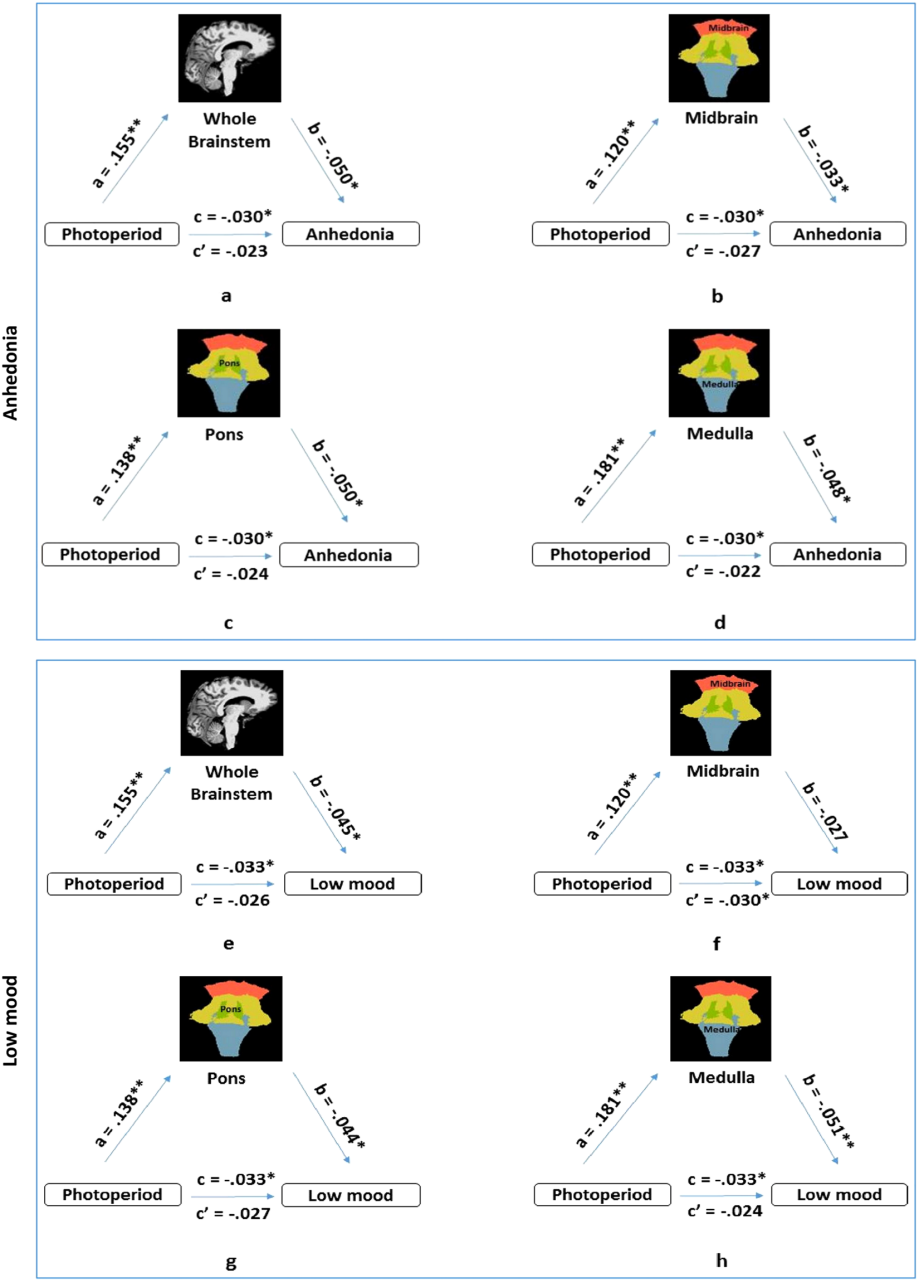
The mediation model demonstrates that the relationship between photoperiod and anhedonia in females was mediated by whole brainstem (**a**), midbrain (**b**), pons (**c**) and medulla (**d**) and the relationship between photoperiod and low mood was meditated by whole brainstem (**e**), pons (**g**) and medulla (**h**), while the relationship between photoperiod and low mood was not meditated by midbrain (**f**). Standardized beta values are shown on the model paths. **a** and **b** included in the model show the direct effects of the relationship between photoperiod and depressive symptoms. **c** and **c’** show the total and the indirect effects of the relationship between photoperiod and anhedonia as well as low mood without and with the mediators respectively. *p < 0.05, **p < 0.001.

A mediation analysis was performed to examine whether brainstem volumes mediate the relationship between photoperiod and depressive symptoms including low mood and anhedonia in females (Fig. 2). Negative binomial regression analysis (corrected for age, ethnicity, living area, Townsend deprivation index and TBV) was used to test path-correlations. Photoperiod and the hypothesised mediator(s) (whole brainstem, midbrain, pons and medulla volumes) were significantly associated (Table 4). In addition, whole brainstem, pons and medulla volumes were significantly related to both low mood and anhedonia, while midbrain volume was significantly related to anhedonia (Table 5). To test whether volume reduced the associations of photoperiod and anhedonia or photoperiod and low mood, whole brainstem, midbrain, pons and medulla volumes were added separately as predictors to negative binomial regression models. We found that: (1) the association between photoperiod and anhedonia was reduced and no longer significant when whole brainstem, midbrain, pons and medulla were included; β = −0.023, CI [−0.053 to 0.006] and p = 0.114 for whole brainstem, β = −0.027, CI [−0.057 to 0.002] and p = 0.066 for midbrain, β = −0.024, CI [−0.054 to 0.005] and p = 0.100 for pons, and β = −0.022, CI [−0.053 to 0.007] and p = 0.127 for medulla, (2) the association between photoperiod and low mood was reduced and no longer significant when whole brainstem, pons and medulla were included, β = −0.026, CI [−0.059 to 0.002] and p = 0.072 for whole brainstem, β = −0.027, CI [−0.060 to 0.001] and p = 0.062 for pons and β = −0.024, CI [−0.057 to 0.005] and p = 0.097 for medulla, while the association between photoperiod and low mood remained significant and did not reduce when midbrain was included, β = −0.030, CI [−0.063 to −0.002] and p = 0.040. Because these results satisfy the requirements of the mediation analysis, we examined whether whole brainstem, midbrain, pons and medulla significantly mediate the relationship between photoperiod and anhedonia, and whether whole brainstem, pons and medulla significantly mediate the relationship between photoperiod and low mood.

To formally test the mediation, we used a bias corrected and accelerated bootstrap method (PROCESS macro in SPSS). The indirect effects were significant (See Table 6) meaning that longer photoperiod days were associated with (1) larger whole brainstem, midbrain, pons and medulla volumes which in turn were related to reporting reduced anhedonia, β = −0.046, CI [−0.114 to −0.027] and p = 0.001 for whole brainstem, β = −0.030, CI [−0.104 to −0.003] and p = 0.037 for midbrain, β = −0.047, CI [0-0.108 to −0.026] and p = 0.001 for pons, and β = − 0.043, CI [−0.093 to −0.019] and p = 0.003 for medulla, and (2) larger whole brainstem, pons and medulla volumes which in turn was related to reporting reduced low mood, β = −0.041, CI [−0.110 to −0.020] and p = 0.005 for whole brainstem, β = −0.040, CI [−0.103 to −0.017] and p = 0.006 for pons, and β = −0.046, CI [−0.100 to −0.023] and p = 0.002 for medulla. The indirect effect of midbrain was not significant on the relationship between photoperiod and low mood, β = −0.023, CI [−0.095 to 0.010] and p = 0.112, suggesting that it was not associated with reporting reduced low mood. When the p-value was Bonferroni corrected (p = 0.006), the indirect effects of the mediators (midbrain, pons, medulla and whole brainstem) on the relationship between photoperiod and anhedonia and low mood (except midbrain) remained significant. To conclude, in females whole brainstem, midbrain, pons and medulla volumes mediate the relationship between photoperiod and both anhedonia and low mood, while midbrain volume mediates the relationship between photoperiod and anhedonia.

**Table 6:** Mediation analyses examining the relationship between photoperiod and both low mood and anhedonia in females via whole brainstem, midbrain, pons and medulla volumes.

	Low mood			Anhedonia		
	b (SE)	Bootstrap CI [LL to UL]	p	b (SE)	Bootstrap CI [LL to UL]	P
Brainstem	−0.0063 (0.002)	−0.0112 to −0.0018	**0.004**	−0.0072 (0.002)	−0.0120 to −0.0027	**0.001**
Midbrain	−0.0027 (0.001)	−0.0062 to 0.0007	0.112	−0.0036 (0.001)	−0.0074 to −0.0001	**0.037**
Pons	−0.0054 (0.002)	−0.0096 to −0.0014	**0.005**	−0.0063 (0.002)	−0.0106 to −0.0025	**0.001**
Medulla	−0.0083 (0.002)	−0.0139 to − 0.0028	**0.001**	−0.0079 (0.002)	−0.0137 to −0.0024	**0.002**

Negative binomial regression coefficients (b), robust standard error (SE), bootstrapping confidence interval (CI), lower level and upper level confidence interval (LL and UL) for the mediation analysis of brainstem substructure volumes on the relationship between photoperiod and depressive symptoms. Significant associations (p < 0.05) are shown in bold.

## Discussion

We have shown that brainstem volumes are associated with photoperiod in humans. Interestingly, we found that in females whole brainstem, pons, and medulla volumes mediated the relationship between photoperiod and both anhedonia and low mood, while midbrain volume mediated the relationship between photoperiod and anhedonia only. No mediation effects for the other depressive symptoms were found in females. No mediation effects were found in males. These findings are the first to demonstrate the mediating effects of brainstem volumes on the seasonal variability of mood and anhedonia.

We also found that photoperiod was associated with depressive symptoms including low mood and anhedonia in females but not in males, where longer photoperiod days were associated with reporting reduced low mood and anhedonia. This however did not remain after Bonferroni correction. This association has been previously reported^4^. Lyall *et al*., study had significantly greater statistical power (n = up to 80,000) which may explain why the association no longer remains significant after correction.

To our knowledge no previous animal or human studies have reported seasonal variations in brainstem volumes with only a small number focusing on the association between photoperiod and the density of serotonergic and dopaminergic neurons and binding transporters in the brainstem, especially midbrain or raphe nuclei as described above^20,21,17,18,30^. However, circadian rhythms are generated and maintained by a neural clock that is regulated by midbrain raphe nuclei in the suprachiasmatic nucleus (SCN)^31^. Therefore, any circadian clock disruptions caused by changes in photoperiod may alter midbrain raphe nuclei which in turn may lead to morphology changes of the brainstem. Our findings support the notion that changes in photoperiod change the brainstem substructure volumes.

The biological mechanisms behind changes in brainstem volumes in mood disorders are still unclear. Previous studies^22–24^ that have shown that individuals with major depressive disorder (MDD) showed increased whole brainstem and midbrain volumes compared to healthy controls. In addition, previous studies^32^ have shown that the echogenicity of the brainstem raphe nuclei is altered in patients with unipolar depressive disorders (UDD) compared to healthy controls. Our finding of a negative association between brainstem volumes and depressive symptoms (low mood, anhedonia and total depressive score) in a large population cohort adds to this evidence.

Further, previous studies^30,33,34^ have suggested that seasonal changes in serotonin (5-hydroxytryptamine; 5-HT) expression, which is mainly synthesized by several nuclei of the midbrain and pons such as dorsal raphe nucleus and locus coeruleus^31^ could be the molecular mechanism that drives this correlation. They found that individuals with seasonal affective disorder showed higher cerebral serotonin transporter binding in winter, but not in summer, compared to healthy controls, and this change in serotonin transporter binding was positively associated with severity of depressive symptoms. Together, these results support the notion of the role of the brainstem in regulating related-mood processing.

In addition, the result of the association of mood or depressive symptoms including low mood and anhedonia with season in only females before correction for multiple comparisons is consistent and supported by several previous studies^4,5,35^ in which females were found to have more hospital admissions due to winter depression and also to report higher prevalence of depression or depressive symptoms during shorter photoperiod days in the winter months compared to males. Little is known about the mechanisms underlining sex-related differences in seasonality of mood though this phenomenon has been widely investigated. One possibility is that the sex-related differences in seasonal variation in mood could be due to the differences in cortico-limbic mood regulation network, which includes the hippocampus, amygdala, prefrontal and anterior cingulate cortices and anterior thalamic nuclei, between males and females^36,37^. Interestingly, the features of subgenual anterior cingulate cortex (sgACC), which have been shown to elevate the metabolic activity in the presence of depression, dysfunction in mood disorders are different between males and females, and females exhibit higher levels of reactivity compared to males^37^. Therefore, it is possible that the corticolimbic network of mood regulation in females is more affected by photoperiod than males, leading to impact the mood status during the year between sexes. Another possibility could be explained by the difference in cortisol hormone and inflammatory stress responses, which have been linked to the prevalence of depression^8,38^. It has been shown that females have greater cortisol and inflammatory stress responses are more sensitive to depressed mood when inflammation is present^39,40^. Together, these studies suggest neuroanatomical and/or hormonal sex-related differences that could be the mechanisms underlining seasonal variation in mood between sexes.

The current study design has three limitations. First, our study was cross sectional in which participants were measured only once rather than at different times over the year, therefore the brainstem volumes measured represent inter-individual variance not change. Making a causal statement about seasonal change of an individual brainstem volume would require a longitudinal study. Second, depressive symptom scores were taken from questions about feelings over the previous two weeks and may be subject to recall bias and also gender biases in reporting mood. Third, we included all data available in the January 2017 brain imaging data release. This means that we included participants who may have medical or psychiatric issues related to their brain such as stroke, Alzheimer’s disease, congenital or acquired structural brain defects.

To conclude, our study is the first to demonstrate that brainstem volumes fully mediate the seasonal variability of depressive symptoms. We further showed that this mediating effect is only present in females. This finding advances our understanding of brainstem morphology and suggests it may be an important neural substrate in the pathophysiology of seasonal mood disorders. This finding adds to the evidence supporting the role of photoperiod on brain structural plasticity which will have implications for future investigations of changes in mood associated with human exposure to variations in natural and artificial light.

## Methods

### Participants

From 2006–2010, 502,655 participants aged 37–73 years were recruited into the UK Biobank cohort. Participants attended one of 22 assessment centres across the UK and completed a range of lifestyle, demographic, health and mood questionnaires, cognitive assessments and physical measures^41^, and subsequently brain imaging at a single centre between 2014 and 2016. More details can be found on the UK Biobank online data showcase (http://biobank.ctsu.ox.ac.uk/crystal/label.cgi). The 10,103 participants aged between 45 and 79 years (mean = 62.4, SD = 7.4) in the January 2017 brain imaging data release were included in this cross-sectional study. Sixty-nine participants were excluded from the study because of issues with their T1 weighted MRI structural images. Out of 10,034 participants, 745 participants were excluded because they did not complete their mood measures in the two weeks prior to the scanning.

All UK Biobank participants gave written, informed consent. UK Biobank received ethical approval from the North West Multi-Centre Research Ethics Committee (11/NW/03820). This research was conducted using the UK Biobank Resource under Application Number 24089 (PI Waiter). All UK Biobank methods were performed in accordance with the UK regulations (https://www.ukbiobank.ac.uk/gdpr/). This work makes use of an open access MRI database of images. As a UK based multicentre trial each of the participating sites are compliant with MHRA guidelines for clinical MRI and participants imaged accordingly.

### Environmental variable (Photoperiod)

Photoperiod in hours of daylight on the day of scan was derived from the latitude and longitude information of the location of residence for each participant. Photoperiod in hours was calculated by subtracting sunset from sunrise on the day of scan.

### Mood variable

Mood outcomes composed of scores reflecting the frequency of low mood, anhedonia, tenseness and tiredness over the two weeks before the assessment. Participants were asked to indicate how often they experience these depressive symptoms including low mood, anhedonia, tenseness and tiredness over the previous two weeks during a computerized touchscreen assessment. They were asked the following questions: (a) “Over the past two weeks, how often have you felt down, depressed or hopeless?” for low mood, and (b)” Over the past two weeks, how often have you had little interest or pleasure in doing things?” for anhedonia, (c) “Over the past two weeks, how often have you felt tense or restless?” for tenseness and (d) “Over the past two weeks, how often have you felt tired or had little energy?” for tiredness. One additional score was a total depressive symptoms score (ranged from 0 to 12) and it was calculated by summing all the scores of the four depressive symptoms. Participants responded with the following: “not at all”, “several days”, “more than half the days”, and “nearly every day”. These responses were coded from 0 to 3 respectively, (that is 0 = “not at all”, 1 = “several days”, 2 = “more than half the days”, and 3 = “nearly every day”). These coded responses were derived directly from the Patient Health Questionnaire (PHQ-9), instrument for depression screening^42^.

### MRI acquisition

MRI scans were acquired using a 3 T Siemens Skyra with a standard Siemens 32-channel RF receive head coil^43^. T1-weighted 3D magnetisation-prepared rapid gradient echo (MPRAGE) images were acquired in the sagittal plane within 5 minutes with these parameters: resolution 1 × 1 × 1 mm, TR = 2000 ms, TI = 880 ms, field of view 208 × 256 × 256 mm, iPAT = 2, superior inferior field-of-view 256 mm^43^.

### Volumetric analysis and segmentation

Volumetric processing and segmentation were performed using a development version of the FreeSurfer v6.0 software package (http://surfer.nmr.mgh.harvard.edu), with brainstem segmentation^44^. We chose FreeSurfer for segmentation due to its good reproducibility in brainstem segmentations compared to other methods^45^. FreeSurfer was used to process the data including averaging volumetric T1 weighted images, motion correction, transformation to Talairach image space, nonuniform intensity normalization for intensity inhomogeneity correction, removal of non-brain tissues using hybrid watershed, and segmentation of subcortical volumetric structures; white matter and deep grey matter^46–48^. FreeSurfer was used to segment the brainstem subfield volumes (medulla oblongata, pons, superior cerebellar peduncle and midbrain). Briefly, 39 MRI scans were manually delineated to highlight the whole brainstem, together with manual labelling of brainstem structures in 10 MRI scans from *in vivo* T1 weighted images (1 mm resolution)^44^. The manual delineation and labelling from *in vivo* scans were combined together to build an atlas of brainstem structures with a new robust Bayesian inference algorithm to detect local variations in MRI contrast. For each subject, volumetric data for these four brainstem structure volumes was calculated using the software’s automatic Bayesian segmentation technique^47,49^. Brain volumes including total brain volume (TBV) and intracranial volume (ICV) were also calculated by FreeSurfer using the Talairach transformation matrix created from the registration of normalisation and MNI atlas^50^. All segmentations for the brainstem were visually checked for errors. No manual interventions were performed on the data.

### Statistical analysis

Statistical analyses were conducted using SPSS version 24, with an alpha for all analyses of p = 0.05. We tested the seasonal pattern of photoperiod (as a continuous measure of day length) and we found that it follows a sinusoidal pattern. No further transforms were applied. To investigate the association of photoperiod with depressive symptoms including low mood, anhedonia, tenseness, tiredness and total depressive symptoms score, a negative binomial regression model was used. Likelihood ratio tests for these depressive scores showed that over-dispersion was greater than 1, i.e. their variance was greater than their mean^51^. We investigated the seasonality of brainstem volumes using a cosinor generalized regression analysis with both sine and cosine functions and month as the time variable^52^. Sine and cosine transformations of the month of scan were calculated using the formulas:

1 $$Sin=(2\,\ast \,\pi (M-1)/12)$$

2 $$Cos=(2\,\ast \,\pi (M-1)/12)$$

where *M* = month of scan (integer number from: 1 to 12). We assessed whether the seasonal pattern of the brainstem is sinusoidal, by comparing a model including sine and cosine month transformations and the covariates of age and TBV with models excluding sine and cosine month transformations. We determined two specific criteria for indicating the significance of seasonality or improved model fit and these were (1) significance of sine and/or cosine (cosinor) terms (p < 0.025), with amplitude significantly greater than zero and (2) lower Akaike Information Criterion (AIC) for the model including the cosinor terms^4,52^. The amplitude of the cosinor model (or curve) was calculated as:

$${\rm{A}}=\sqrt{{\beta }^{2}+{\gamma }^{2}}$$

where β *and γ* are cosine and sine generalized regression coefficients respectively. Finally, the Acrophase (ϕ; peak of cosinor model) in month of scan was calculated from:

$$\phi =12\,\ast \,\frac{{\tan }^{-1}({\rm{\gamma }}/{\rm{\beta }})}{2\,\ast \,{\rm{\pi }}}+1$$

Pearson (bivariate) correlations between photoperiod and brainstem substructure volumes as well as age, and total brain volume (TBV) were performed, with significance levels Bonferroni-corrected for multiple comparisons and set at p = 0.003. To investigate the predictability for each of these independent variables for the brainstem subfield volumes, single linear regression models for each were created. There were significant correlations between brainstem subfield volumes and age, total brain volume, ethnicity, living area (urban or rural) and Townsend deprivation index, therefore, these predictors were included as covariates in a multiple regression model. For the mediation analysis, negative regression analysis was used here to test whether brainstem or substructure volumes mediate the relationship between photoperiod and low mood as well as anhedonia. Eight separate mediation analyses were conducted to examine whole brainstem, midbrain, pons and medulla volumes as mediators in the relationship between photoperiod and both anhedonia and low mood, after controlling for the relevant covariates mentioned above. The mediation analysis applied here used the standard three-variable path model^53^, and a bootstrapping test (with 5000 samples to compute the 95% confidence interval) for the statistical significance of the model using the “PROCESS” method^54^ in SPSS version 24.

## Data Availability

The datasets processed and analysed during the current study are available from the online open access UK Biobank repository (https://www.ukbiobank.ac.uk/). This research was conducted under the UK Biobank Resource under Application Number 24089 (PI Waiter).
